# Lipidosterolic Extract of Serenoa Repens Modulates the Expression of Inflammation Related-Genes in Benign Prostatic Hyperplasia Epithelial and Stromal Cells

**DOI:** 10.3390/ijms140714301

**Published:** 2013-07-10

**Authors:** Nanor Sirab, Grégoire Robert, Virginie Fasolo, Aurélien Descazeaud, Francis Vacherot, Alexandre de la Taille, Stéphane Terry

**Affiliations:** 1 INSERM, Unité 955, Equipe 7, Créteil F-94000, France; E-Mails: sirabnanor@gmail.com (N.S.); gregoire.robert@chu-bordeaux.fr (G.R.); 2 CHU de Bordeaux, Service d’urologie, Université Bordeaux Segalen, Bordeaux F-33076, France; 3 Qiagen Marseille, Marseille F-13288, France; E-Mail: fasolo@ipsogen.com; 4 Hôpital Dupuytren, CHU de Limoges, Service d’urologie, Limoges F-87000, France; E-Mail: aureliendescazeaud@gmail.com; 5 Faculté de Médecine, Université Paris Est Créteil, Créteil F-94000, France; 6 AP-HP, Hôpital H. Mondor–A. Chenevier, Service d’urologie F-94000, France; 7 Institute Curie, Centre de Recherche, CNRS UMR3244, Paris F-75248, France

**Keywords:** benign prostatic hyperplasia, inflammation, phytotherapy, *Serenoa repens*, gene expression analysis

## Abstract

Despite the high prevalence of histological Benign Prostatic Hypeplasia (BPH) in elderly men, little is known regarding the molecular mechanisms and networks underlying the development and progression of the disease. Here, we explored the effects of a phytotherapeutic agent, Lipidosterolic extract of the dwarf palm plant *Serenoa repens* (LSESr), on the mRNA gene expression profiles of two representative models of BPH, BPH1 cell line and primary stromal cells derived from BPH. Treatment of these cells with LSESr significantly altered gene expression patterns as assessed by comparative gene expression profiling on gene chip arrays. The expression changes were manifested three hours following *in vitro* administration of LSESr, suggesting a rapid action for this compound. Among the genes most consistently affected by LSESr treatment, we found numerous genes that were categorized as part of proliferative, apoptotic, and inflammatory pathways. Validation studies using quantitative real-time PCR confirmed the deregulation of genes known to exhibit key roles in these biological processes including *IL1B*, *IL1A*, *CXCL6*, *IL1R1*, *PTGS2*, *ALOX5*, *GAS1*, *PHLDA1*, *IL6*, *IL8*, *NFkBIZ*, *NFKB1*, *TFRC*, *JUN*, *CDKN1B*, and *ERBB3*. Subsequent analyses also indicated that LSESr treatment can impede the stimulatory effects of certain proinflammatory cytokines such as IL6, IL17, and IL15 in these cells. These results suggest that LSESr may be useful to treat BPH that manifest inflammation characteristics. This also supports a role for inflammation in BPH presumably by mediating the balance between apoptosis and proliferation.

## 1. Introduction

The histological definition of benign prostatic hyperplasia (BPH) refers to an overgrowth and increased proliferation of the epithelial and stromal components of the transition zone, and of the peri-urethral area of the prostatic gland. The prevalence of histological BPH is over 70% at 60 years of age and over 90% at 70 years of age [1]. Although androgen and estrogen may contribute to the development of histological BPH, the molecular mechanisms leading to pathogenesis of BPH remain poorly understood [2].

From recent *in vitro* and *in vivo* studies, the notion emerged that chronic prostatic inflammation (CPI) could play a major role in the development of BPH [3–5]. The correlation between CPI and hypertrophic prostate was first reported in 1968 and was extensively described since the early 1970s [6]. In 2005, a sub-group analysis of the MTOPS (Medical Therapy of Prostatic Symptoms) study confirmed that CPI could be associated with a higher prostate volume and an increased risk for BPH complication such as acute urinary retention [7]. The REDUCE (REduction by DUtasteride of prostate Cancer Events) study, reported in 2008, confirmed a link between CPI and the International Prostate Symptom Score (IPSS) score [8]. Recently, we examined potential relationships between BPH and CPI, and found that CPI associated with a higher IPSS score (21 *vs*. 12) and a larger prostate volume (77 *vs*. 62 cc) [4]. Thus, inflammation may represent a promising target in BPH management. Non-steroidal anti-inflammatory treatments (NSAIDs) and cyclooxygenase-2 (COX-2) inhibitors have shown positive effects on the lower urinary tract symptoms (LUTS) attributable to BPH [9,10]. However, these therapeutic agents may not be adapted to all/most BPH patients due to their gastro-intestinal and cardiovascular side effects.

The lipidoSterolic extract of the American dwarf palm plant, *Serenoa repens* (LSESr), has been used for the last 25 years for the treatment of LUTS suggestive of BPH (permixon). LSESr is essentially made of a complex mixture of fatty acids of which the free forms account for a mean of 83.5 g/100 g of extract [11]. This mixture includes oleic, lauric, myristic, linoleic, and palmitic acids. LSERs also contains a small proportion of phytosterols, aliphatic alcohols, and polyprenic compounds.

Several mechanisms of action have been proposed for this phytotherapeutic agent, including an inhibitory effect directed to 5α-reductase [11–13], anti-estrogenic effect [14], and anti-proliferative/pro-apoptotic action mediated by growth factors inhibition [13–17]. An anti-inflammatory effect has also been suggested in various settings, LSESr potently antagonizing the cyclooxygenase-2 (COX-2) and 5-lipoxygenase (5-LOX) metabolites production or suppressing the expression of inflammatory mediators such as MCP-1 and VCAM-1 [18–21]. Here, we describe our experience in testing benign human prostate epithelial and stromal cell models for their responsiveness to LSESr by analyzing LSESr-induced changes of gene expression profiles and cell behavior in cultures.

## 2. Results

### 2.1. LSESr Affects Cell Viability in Cultured Human Epithelial and Stromal Cells Derived from Benign Prostatic Hyperplasia (BPH)

**Figure 1:**
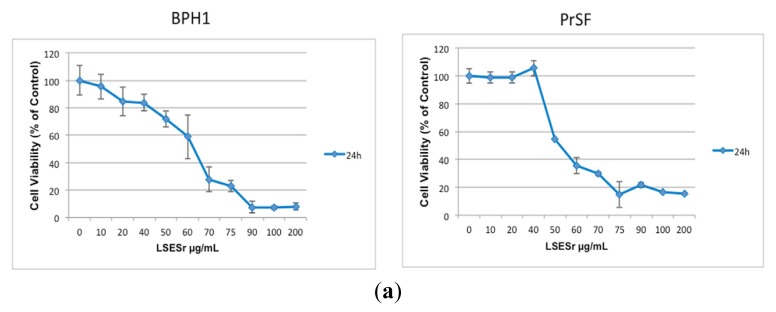

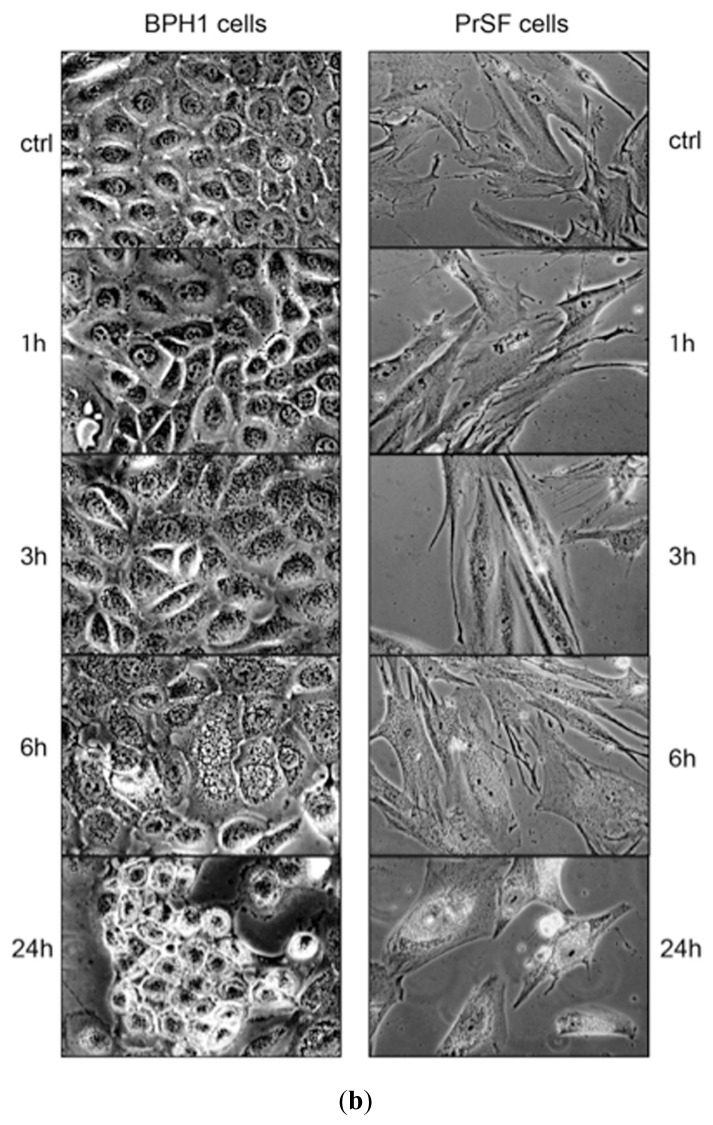
(**a**) MTT assay showing dose ranging (10 to 200 μg/mL) effect of lipidosterolic extract of *Serenoa repens* (LSESr) at 24 h on the cell viability of BPH1 immortalized epithelial cell line (left) and primary stromal fibroblasts (PrSF) primary culture of stromal cells (right). The mean value of the optical density (OD) measured in the control condition (0) was defined as 100% survival with other conditions reported as a percent of the control. Error bars show standard error of the mean; (**b**) Microscopic observation of BPH1 epithelial cells (left) and PrSF stromal cells (right) after 1 h, 3 h, 6 h, and 24 h exposure to LSESr as compared to untreated cells (0 h). Phase contrast photomicrographs (40×) show the accumulation of vesicles in the cytoplasm of both cell types after 3 h of exposure to LSESr. In this experiment, the final concentration of LSESr is 60 μg/mL for epithelial cells and 50 μg/mL for stromal cells.

We first evaluated the effect of LSESr on cell viability of the available immortalized BPH1 human prostate epithelial cells, as well as in primary stromal fibroblasts (PrSF) isolated in our site. Cells were exposed to LSESr for 24 h with doses ranging from 10 to 200 μg/mL, and viability was assessed using MTT assay. In these conditions, LSESr showed a dose-dependent cytotoxic effect with cells showing almost 100% decrease of cell viability when exposed to the 100 μg/mL dose or above (Figure 1a). Stromal and epithelial cells respond similarly to LSESr. The estimated 50% lethal concentration (LC50) was 60 μg/mL for BPH1 cells and 50 μg/mL for PrSF. Since drastic effects were evident at 24 h following treatment, we wondered whether the cells show any early morphological changes after exposure to LSESr. Light microscope analysis revealed cytoplasmic vesicles in BPH epithelial and stromal cells, as early as three hours of LSESr supplementation and increasing with time (Figure 1b).

### 2.2. Gene Expression Profiling of BPH Epithelial and Stromal Cells Treated with LSESr

#### 2.2.1. LSESr Induces Changes in Gene Expression Pattern in BPH Epithelial and Stromal Cells

We explored the dynamic effect of LSESr on gene expression profiles of stromal and epithelial BPH cells. Using Affimetrix human U133 Plus 2.0 Arrays, gene expression profiles were successfully obtained from BPH1 epithelial cell line and PrSF stromal cells exposed to LSESr for one, three, or six hours, and compared to untreated control cells.

**Figure 2:**
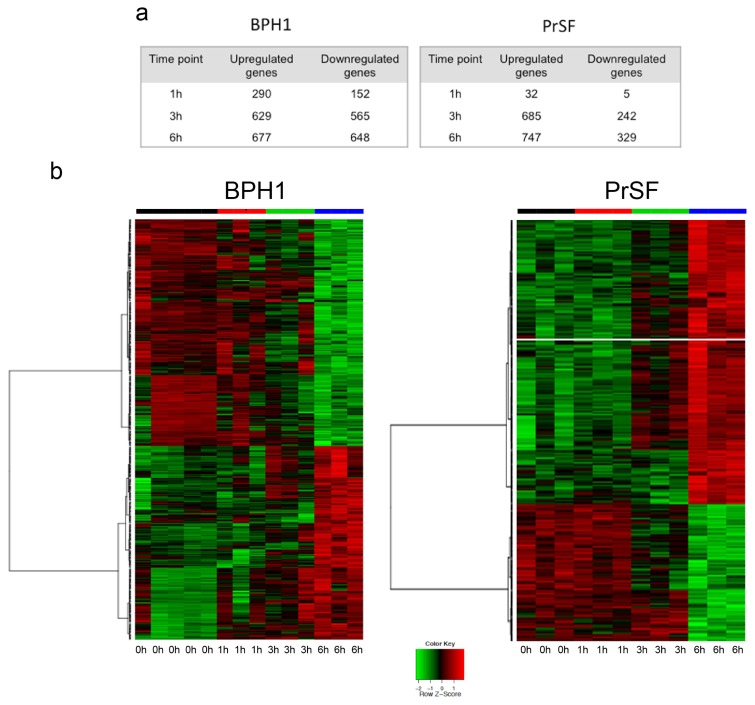
Microarray profiling of gene expression in control (0 h) and LSESr treated BPH epithelial (left) and stromal (right) cells. BPH cells are treated in three replicates for 1, 3, and 6 h by LSESr (60 μg/mL for BPH1, 50 μg/mL for PrSF). (**a**) Differential analysis representing the number of up/down-regulated genes at 0 h, 1 h, 3 h, and 6 h; (**b**) Heat maps demonstrating the relative level of differentially expressed genes after 6 h exposure to LSESr. An FDR adjusted *p*-value represents the significance of the enrichment. Only annotations with a significant FDR adjusted *p*-value of <0.05 are shown.

A first explorative analysis by hierarchical clustering incorporating genes most significantly deregulated indicated substantial differences between treated and untreated cells, and between different time points (not shown). In both cell types, clustering of deregulated genes demonstrated that three-hour and six-hour exposures were more closely related, while separated from untreated and one-hour exposure. On further analysis, we inspected for differentially expressed genes in the different time points relative to untreated cells (Table S1a,b). One hundred forty of the differentially expressed genes were in common between both cell types (Table S1c), however only a few seemed similarly up-or down-regulated at the respective time points such as *GAS1* (growth arrest-specific 1) and *CDKN1B* (cyclin-dependent kinase inhibitor 1B (p27, Kip1)). This information argues for distinct cell responses in the two models. The maximum number of differentially expressed genes in both cell types was observed after six hours of LSESr treatment (Figure 2a). Similarly, in the epithelial cells, 677 genes were up-regulated and 648 were down-regulated upon 6 h exposure to LSESr (Figure 2b). In the stromal cells, 747 genes were up-regulated and 329 genes were down-regulated after six hours of exposure to the LSESr (Figure 2a,b).

#### 2.2.2. Changes in Apoptosis, Proliferation, and Inflammation Related Processes Revealed by Gene Ontology (GO) Analysis

**Figure 3:**
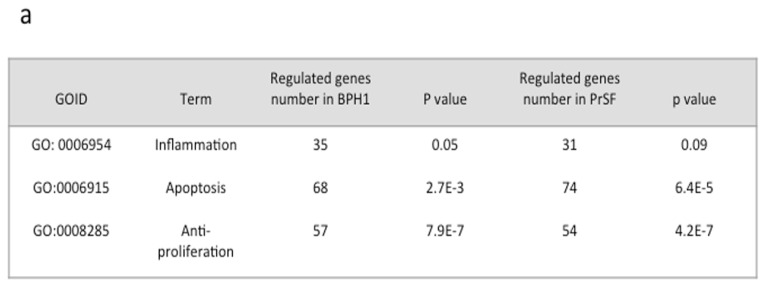

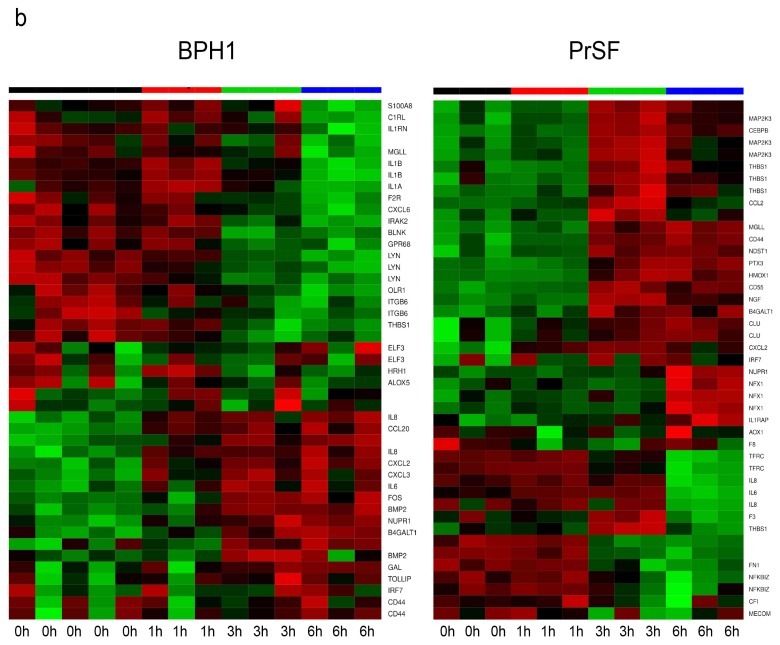
Gene Ontology (GO) analysis. (**a**) DAVID analysis for differentially regulated genes, which belong to the categories of inflammation, apoptosis and regulation of proliferation in at least one time point. Gene Ontology (GO) based annotation was used to perform functional enrichment analysis using DAVID tools; (**b**) Heat maps of significantly differentially expressed genes involved in the inflammation process in BPH epithelial cells (**left**) and PrSF cells (**right**) treated by LSESr at different time-points as compared to control. Genes significantly up-regulated are in red and down-regulated are in green. Black bar is for control (0 h), red for 1 h, green for 3 h, and blue for 6 h of exposure to LSESr.

We then wondered whether the gene expression patterns in epithelial and stromal cells treated by LSESr are related to any particular biological function. To this end, we applied GSA (gene set analysis) [22] to investigate overrepresented pathways among genes with up- or down-regulation in treated cells. We found that the majority of genes deregulated by LSESr exposure are involved in several biological processes as defined by Gene Ontology (GO) databases. This included cellular metabolism, cell cycle and differentiation, apoptosis, organ morphogenesis, hormone secretion, angiogenesis, phosphorylation, signal transduction, cellular responses to pathogens, and external stimulus (Table S1d,e). Furthermore, using the DAVID functional annotation, we found that among functional terms that gather several of these processes, apoptosis, proliferation, and inflammation terms were greatly affected, although the inflammation group failed to demonstrate statistical significance in the surveyed stromal cells (Figure 3a). Overall, 160 genes and 155 genes were found in these categories for epithelial and stromal cells, respectively. Other biological processes potentially associated with inflammation were consistently perturbed in BPH1 cells (e.g., leukocytes chemotaxis, leukocyte migration, cytokine production, immune system development, regulation of response to stimulus) (Table S1d) and in PrSF cells to a lesser extent (e.g., cytokine production, leukocyte activation, regulation of I-kappaB kinase/NF-kappaB cascade) (Table S1e).

Of the proliferation markers markedly affected after six hours of treatment in BPH1 epithelial cells, *PHLDA1* (pleckstrin homology-like domain, family A, member 1) was down-regulated (Fold Changes (FC) = −1.1), whereas *GAS1* and *DEDD* (death effector domain containing) expressions were augmented (FC = 1.45 and 0.77, respectively) (Figure S1a, Tables S1f,g and 1). In PrSF stromal cells, a significant decrease in *ERBB3* expression (v-erb-b2 erythroblastic leukemia viral oncogene homolog 3 (avian)) and *JUN* (jun proto-oncogene) was observed as compared with untreated cells (FC = −0.97 and −0.76, respectively). Moreover, genes well known for their anti-proliferative and/or pro-apoptotic effect, such as *CDKN1B* (FC = 0.84) and *GAS1* (FC = 0.73), were up-regulated in this cell type upon exposure to LSESr (Figure S1b, Tables S1h,i and 1).

#### 2.2.3. LSESr Perturbs Expression of Inflammation-Related Genes in BPH Epithelial and Stromal Cells

We then sought to appreciate further the effect of LSESr on inflammation-related genes by analyzing their expression at the different time points. Generated heatmaps showed a marked deregulation of the genes in both epithelial and stromal cells following LSESr exposure (Figure 3b). Among the genes affected in epithelial cells treated for six hours, we observed substantial down-regulation (−1.67 ≤ FC ≤ −0.58) of *IL1A* (interleukin 1, alpha), *IL1B* (interleukin 1, beta), *CXCL6* (chemokine (C-X-C motif) ligand 6), *ALOX5* (arachidonate 5-lipoxygenase), *IL1R1* (interleukin 1 receptor, type I), and *PTGS2* (prostaglandin-endoperoxide synthase 2, commonly referred to as COX2), while *NFKBIA* (nuclear factor of kappa light polypeptide gene enhancer in B-cells inhibitor, alpha) was up-regulated in this setting (Figure 3b, Tables 1 and S1a,j).

With regards to stromal cells, genes such as *IL6* (interleukin 6), *IL8* (interleukin 8), *NFKBIZ* (nuclear factor of kappa light polypeptide gene enhancer in B-cells inhibitor, zeta), as well as *TFRC* (transferrin receptor (p90, CD71) were found as being consistently down-regulated (−1.69 ≤ FC ≤ −1.1) after six hours treatment with LSESr (Figure 3b, Tables 1 and S1b,k). These results suggest that LSESr treatment in both BPH cell types modulated the expression of genes involved in inflammation, cell growth, and survival pathways.

**Figure 4:**
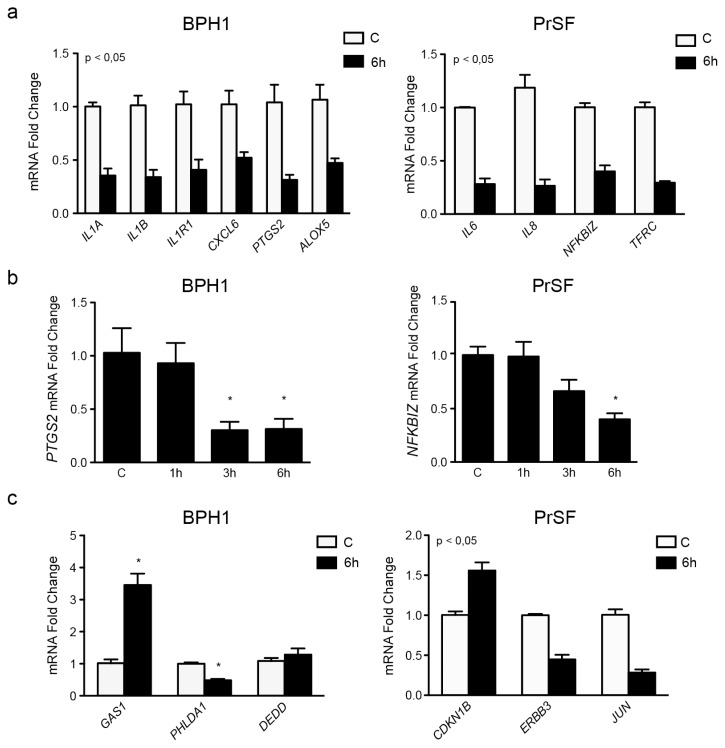
Real Time PCR (RT-PCR) measurements confirming the results of the Microarray study. (**a**) qRT-PCR analysis for mRNA expression of some inflammatory markers in BPH1 cells (left): *IL1B*, *IL1A*, *IL1R1*, *CXCL6*, *ALOX5*, *PTGS2*, and *ALOX5* and in PrSF cells (right) *IL6*, *IL8*, *NFKBIZ*, and *TFRC*; (**b**) RT-PCR showing a time-dependent regulation of mRNA expression by LSESr. The transcript expression of *PTSG2* in BPH1 cells (left) was decreased significantly after 3 and 6 h of LSESr supplementation. *NFKBIZ* down-regulation in PrSF cells was significant only after 6 h; (**c**) qRT-PCR analysis for mRNA expression of Apoptosis and cell cycle related genes (*GAS1*, *CDKN1B*, *PHLDA1*, *ERBB3*, *DEDD*, and *JUN)*. Bars, SD for three experiments, each performed in duplicate; *p < 0.05, **p < 0.01.

To ascertain this finding, we then chose a set of genes from the previous lists, and their mRNA expression was inspected by real time RT-PCR for deregulation upon six hour of treatment. Ten genes were selected based upon their putative role in the inflammation pathways (*IL1A*, *IL1B*, *CXCL6*, *IL1R1*, *ALOX5*, and *PTGS2* for epithelial cells, *IL6*, *IL8*, *NFKBIZ*, and *TFRC* for stromal cells) and six additional genes were selected for their potential involvement in apoptosis/proliferation pathways (*DEDD*, *PHLDA1*, *GAS1*, *CDKN1B*, *ERBB3*, and *JUN*). The results obtained from these experiments were highly consistent with the microarray data, thus validating the previous experiments (Table 1). As shown in Figure 4, the mRNA expression of 15 out of 16 selected genes was significantly affected by the exposure to LSESr, including all the inflammatory related genes presenting with pronounced decrease in expression detected at the six-hour time point (Figure 4a). We also noted an apparent time-dependent regulation for some of these genes including *PTGS2* (COX2) in BPH1 cells, and *NFKBIZ* in PrSF cells (Figure 4b). Significant increase in the expression levels of *GAS1* (FC = 1.79) and *CDKN1B* (FC = 0.64), genes specific for cell growth suppression was also detected in BPH1 and PrSF, respectively (Figure 4c). In contrast less remarkable expression changes were found for *DEDD*, although we could note a trend to augmented expression as expected from the microarray results.

### 2.3. LSESr Impedes the Effects of Pro-Inflammatory Factors on Proliferation in BPH Epithelial and Stromal Cells

**Figure 5:**
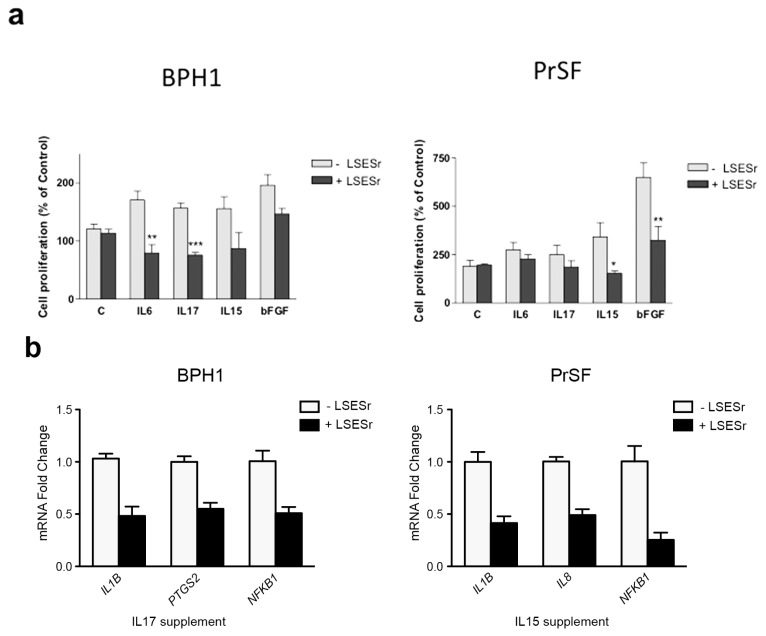
Effects of LSESr in BPH epithelial and stromal cells stimulated by inflammatory factors. (**a**) BrdU incorporation assay investigating cell proliferation index. Cells were treated with 10 ng/mL of recombinant human IL6, IL17, IL15, or bFGF for 24 h in presence or absence of LSESr; (**b**) qRT-PCR analysis for mRNA expression of some inflammatory markers in epithelial cells stimulated by IL17 (**left**), or in stromal cells stimulated by IL15 (**right**) in the presence or absence of LSESr. In BPH1 cells, *PTGS2*, *IL1B* and *NFkB1* were down-regulated by LSESr.

Previous findings are consistent with an inhibitory role of LSESr on the intrinsic inflammatory gene expression pattern in cultures of BPH epithelial and stromal cells. It is worth noting that chronic inflammation in BPH is assumed to mediate epithelial/stromal interactions. Thus, a variety of cytokines and growth factors may be released by inflammatory cells, presumably regulating the differentiation and the growth of stromal and epithelial BPH cells. In an attempt to further appreciate the anti-inflammatory activity of LSESr, we examined cell proliferation in cultures of BPH-1 and PrSF cells that were supplemented with bFGF (basic Fibroblast Growth Factor) or cytokines (10 μg/mL) in the presence or absence of LSESr, at concentration below LC_50_ in order to limit its potential effects on apoptosis. A set of interleukins defined as pro-inflammatory in BPH including IL6, IL15, and IL17 were selected for these experiments. In BPH1 cells, adjunction of these pro-inflammatory signals alone seemed to stimulate cell proliferation as assessed by BrdU incorporation (Figure 5a, left). The addition of 50 μg/mL of LSESr to the culture medium of the stimulated cells resulted in a seeming decrease of the proliferation index. Statistical significance was achieved in cells stimulated by IL6 and IL17. With regards to stromal cells, bFGF and IL15 induced cell proliferation relative to control cells and LSESr 40 μg/mL was sufficient to significantly impair the proliferation in cells supplemented with IL15 and bFGF (Figure 5a, right).

These results suggested that the inflammatory response and proliferation processes might be linked in BPH cells, at least to some extent, and that LSESr can disturb this link. To further test this possibility, we assessed the transcript expression of different inflammatory-related genes in cultures of BPH1 or PrSF cells supplemented with IL17 and IL15, respectively, in the presence or absence of LSESr. In these conditions, we found that LSESr treatment down-regulated genes such as *PTGS2*, *IL1B*, and *NFKB1* in the epithelial cells receiving IL17 supplement (Figure 5b). Expression of *IL8*, *IL1B*, and *NFKB1* was likewise reduced in stromal primary cells receiving IL15 supplement. These findings suggest that LSESr might suppress inflammation/proliferation processes mediated by pro-inflammatory factors in both BPH epithelial and stromal cells.

### 2.4. Discussion

The Lipidosterolic extract of *Serenoa repens* (LSESr) is the most widely studied and prescribed phytotherapeutic agent for the treatment of lower urinary tract symptoms (LUTS) in BPH, mainly because of positive comparisons to α-blockers and 5α-reductase inhibitors [12]. However, its precise mechanisms of action remain to be elucidated. In this study, we explored the consequences of LSESr treatment on gene expression patterns in cultures of BPH epithelial and stromal cells, which represent the two predominant cell contingents in BPH. To our knowledge, this study is the first to report gene expression profiling in BPH cells treated by LSESr. We confirmed the anti-inflammatory role of LSESr and provide new evidence that LSESr affects various inflammatory genes and pathways with putative roles in BPH pathology [3]. The pathogenesis of BPH is age-related and dependent upon the presence of androgens [2]. The prevalence of BPH increases with age, whereas levels of circulating androgens generally decline. Thus, although androgen may play a permissive role in BPH, other molecular processes are certainly involved in the initiation and development of BPH. The chronic prostatic inflammation (CPI) might be a good candidate in this regard [3,4,23]. As a corollary, it would appear at first quite relevant to treat the urinary symptoms of BPH with anti-inflammatory agents, such as NSAIDs and COX-2 inhibitors [10,24,25]. However, these drugs are not recommended for a chronic condition such as BPH because of their important side effects. Therefore, the interest rises for using phytotherapeutic agents in LUTS treatment as substitute of synthetic molecules. LSESr plant extract, which is believed to have certain anti-inflammatory effects, has been prescribed for more than 25 years without any major side effect being reported. Vela Navarette *et al*., first described LSESr effect on prostate inflammatory status in a clinical study showing a significant reduction in the number of B-lymphocytes (58.2 *vs*. 91.4; *p* = 0.097) and other immune response markers (TNFα and IL-1β) after treatment [21]. In our study, we used two suitable models derived from BPH specimens to further investigate the regulatory mechanisms underlying BPH and to study the immediate effects of LSESr on the two major components of this pathology. Our gene expression survey indicated that LSESr greatly influences genes belonging to immune response, apoptosis and cell proliferation pathways. Such deregulation was hardly detectable after one hour of treatment while the maximum number of deregulated genes was observed after six hours of treatment. Also of interest is that accumulation of cytoplasmic vesicles was apparent after three hours of LSESr exposure suggesting rapid entry of LSESr into cells along with potential changes in the lipid composition of membranes.

Notably, multiple inflammation functional pathways, such as Cytokines family, Glucocorticoid/PPAR signaling, MAPK signaling, TNF superfamily, and COX/LOX pathways, seemed to be affected by LSESr treatment. Some of these genes encode for molecules assumed to be key mediators in the inflammation process such as *IL6*, and *IL8* in stromal cells [26–29], or *IL1A*, *IL1B*, and their receptor *IL1R1* in epithelial cells. Earlier work suggested that LSESr could perturb the production of COX-2 and 5-LOX metabolites [18]. Our microarray study further supports the down-modulation of *PTGS2* (which encodes the protein COX-2) and *ALOX5* (which encodes the protein 5-LOX) in the epithelial cells. *CXCL6*, which is also down-regulated in LSESr-treated BPH1 cells, encodes a protein with pro-angiogenic properties and chemoattractant for neutrophils [30]. Interestingly, an anti-inflammatory effect of LSESr was proposed in previous work focusing on the inflammation mediators MCP-1/CCL2 and VCAM-1 [20] in various cell populations including neutrophils, vascular endothelial and myofibroblast cells. *TFRC* has been proposed as a tumor biomarker in a wide spectrum of malignancies, including B-cell lymphoma, pancreatic, esophageal, cervical as well as prostate cancers [31–36]. In macrophages, its expression is induced following pro-inflammatory signals *via* an NF-κB-dependent mechanism that also involves HIF-1 activation [37]. Of further interest is that among the genes consistently perturbed by LSESr treatment, at least seven were well-known transcriptional targets of NF-κB (*IL1B*, *IL1A*, *IL6*, *IL8*, *IL1RAP*, *PTGS2*, *NFKBIZ*). In many diseases and cell types, the nuclear factor of kappa beta (NF-κB) has been incriminated as a master regulator of biological processes especially cell growth, proliferation and inflammation [38–40]. Clearly, future studies should be performed to gain further insights into this effect.

BPH is defined by an exacerbated growth of stromal and epithelial cells of the prostate consecutive to alterations in cell proliferation, differentiation and apoptosis. Our data confirmed previous suggestions for the anti-proliferative and pro-apoptotic actions of LSESr [15,17], and in a manner that seems closely related to an anti-inflammatory response in BPH cells. Interleukin-mediated growth of BPH cells was found to be affected by LSESr treatment. Inflammation may disrupt the intricate balance between cell growth and cell death homeostasis resulting in increased proliferation, and apoptosis reduction [3,23,30]. Thus, when testing new compounds in the setting of BPH, one should consider cross-modulations occurring between these three biological processes. Although, as yet unvalidated experimentally, our MicroArray analysis herein reveals that multiple metallothionein (MT) members were consistently up-regulated in both the PrSF and BPH1 cells treated with LSESr (Table S1a–c). This could be especially relevant biologically since MTs proteins are assumed to regulate a number of cellular processes including gene expression, apoptosis, proliferation, differentiation, and inflammation [41,42]. Clearly, this finding gives reason to consider whether these factors might play a role in BPH or in the response to LSESr.

Collectivity, our findings provide support for the view that the inflammatory response and proliferation/apoptosis pathways might be related in BPH pathogenesis, and LSESr interferes with these processes. Although promising, these findings remain to be clarified in the *in vivo* setting. Existing animal models could help in this respect [43–45]. To confirm the anti-inflammatory effect of the LSESr in BPH patients, it would also be necessary to collect prostatic tissue before and after treatment to perform the same microarray analysis. Such investigations are facing ethical concerns partly due to the invasiveness of the prostatic biopsies. As a surrogate, a placebo randomized clinical trial evaluating the effects of LSESr on non-invasive biomarkers could be proposed. Such clinical studies may suffer from the lack of non-invasive biomarkers for optimal patient stratification and monitoring of disease progression. Other than PSA, which seems specific for prostate tissue, but not for BPH, there is currently no reliable biomarker available for this disease. A few candidates are promising such as C-reactive protein, IL6, and low sTNF-RII associated with increased risk of BPH [5,28]. Interestingly, IL8 and CXCL6 appearing in this study were previously proposed as potential predictive markers of BPH [29,30,46]. Moreover, *PTGS2*, *TFRC*, *JUN*, *ILA*, *ILB*, *NFKBIZ*, *CDKN1B*, and *GAS1*, although not considered as prostate specific genes, can all achieve substantial levels in BPH cells. This supports the need for further evaluation.

## 3. Experimental Section

### 3.1. Cell Culture

Human hyperplasic cell line BPH1 was purchased from the German Collection of Microorganisms and Cell Cultures (DSMZ, Braunschweig, Germany). Cells were maintained in RPMI 1640 culture medium (Life Technologies, Cergy-Pontoise, France) enriched by 20% fetal bovine serum (FBS), 20 ng/mL testosterone, 1% insulin-transferrin-selenium, 100 units/mL penicillin and 100 μg/mL streptomycin.

Primary cultures of BPH stromal cells were obtained from surgical BPH tissues by collagenase digestion. Briefly, the prostatic tissue was washed with HBSS and diced into small pieces. The diced tissue was incubated for 6 h at 37 °C in MCDB131 medium (Life Technologies) containing 300 U/mL collagenase (Roche Diagnostics, Manheim, Germany), 50 μg/mL DNase I (Roche Diagnostics, Meylan, France), 0.03% pronase and 0.08% trypsine inhibitor (Sigma, St. Louis, MO, USA). After digestion, stromal fibroblasts/myofibroblasts were separated from epithelial cells by differential centrifugation. Supernatant enriched with stromal cells was then washed by HBSS and resuspended in MCDB131 supplemented with L-glutamine, penicillin/streptomycin, 2.5 μg/L fungizone, and 10% FBS. The separated cells, primary stromal fibroblasts (PrSF), were then incubated at 37 °C in 5% CO_2_. The cells were serially passaged two to five times before being used in the experiments to eliminate any contamination by epithelial cells.

### 3.2. Lipidosterolic Extract of *Serenoa repens*

Lipidosterolic extract of *Serenoa repens* (LSESr) (Permixon^®^, Pierre Fabre Médicament, Castres, France) was dissolved before use at 100 mg/mL in pure ethanol to obtain a homogeneous solution.

### 3.3. Cell Viability Assay

Cell viability was assessed by using 3-(4,5-Dimethylthiazol-2-yl)-2,5-diphenyl-2*H*-tetrazolium bromide (MTT) solution (Sigma-Aldrich, St. Louis, MO, USA) to study the cytotoxicity of LSESr on both epithelial and stromal cell types. Briefly, cells were seeded in 96-well plates at a density of 10^4^ cells/well. Twenty-four hours later, cells were treated with increasing concentrations of LSESr ranging from 10 to 200 μg/mL and were incubated for 24 h. MTT was added with a final concentration of 1 mg/mL and incubated for 2 h at 37 °C. MTT formazan crystals were dissolved in isopropanol and the optical density (OD) was measured at 540 nm wavelength using a microplate reader multiskan (Thermo Fisher Scientific, Waltham, MA, USA). The cell viability was expressed as the percentage of the OD in cells with LSESr treatment *versus* untreated control. The 50% lethal concentration (LC50) of LSESr was determined from the obtained graphs as the concentration, which reduced cell survival to 50%. The results were generated from three independent experiments and each experiment was performed in triplicate.

### 3.4. Cell Proliferation Assay

Proliferation assay using a BromodeoxyUridine (BrdU) Cell Proliferation Kit (Perkin-Elmer, Courtaboeuf, France) was performed to assess the effect of LSESr on proliferation index in both cell types. Cells were seeded in 96-well plates in 4 replicates at a density of 10^4^ cells/well, allowed to attach for 24 h. Then, cells were treated with various recombinant human interleukins: IL6, IL17, IL15 purchased from R&D Systems (Minneapolis, MN, USA) and basic Fibroblast growth factor (bFGF) (kindly provided by Prof. Jean Delbé) for 24 h at a final concentration of 10 ng/mL alone or in combination with LSESr. BrdU labeling solution was added to the treated cells and incubated for 18 h. After removal of the culture medium, the cells were fixed, permeabilized, and the DNA was denatured. Anti-BrdU-Eu antibody solution was added and the signal was developed with tetramethyl-benzidine solution in darkness. Europium fluorescence emission was measured to determine the proliferation index of the cells using Wallac victor^3^ 1420 Multilabel counter (Perkin-Elmer, Courtaboeuf, France).

### 3.5. Microarray Hybridization and Analysis

Epithelial and stromal cells of BPH were seeded in flasks. Twenty-four hours later, cells were treated with LSESr at a concentration equal to the 50% lethal concentration. Cells were then incubated for 1, 3, and 6 h before RNA extraction. The experiments were performed in three replicates and untreated cells were used as control.

Total RNA was extracted from cell cultures using TRIzol reagent and Purelink RNA extraction kit (Life Technologies, Grand Island, NE, USA). RNA quality was assessed to have an RNA integrity number (RIN) of at least nine using an Agilent BioAnalyzer (Agilent Technologies, Santa Clara, CA, USA). Total RNA was further purified and treated as described by the manufacturer of the microarrays (Affymetrix, Cleveland, OH, USA), and hybridized on Affymetrix GeneChip Human genome U133 Plus 2.0 Arrays (Affymetrix, Cleveland, OH, USA).

Each microarray contained 54,000 probe sets that included a total of 47,000 genes. Target preparation, hybridization, and signal acquisition were done according to standard Affymetrix protocols. Each sample and hybridization underwent a quality control evaluation by using internal housekeeping genes (*beta-actin* and *GAPDH*), *poly-A* spike-in controls (RNAs *Lys*, *Phe*, *Thr,* and *Dap* from *Bacillus subtilis*) and hybridization controls (RNAc *bioB*, *bioC*, *bioD*, and *cre* from *Escherichia coli*), *etc.* Quality control was considered good when profiles and detected signals from *poly-A* spike-in controls, 3′/5′ expression ratio of *beta-actin* and *GAPDH,* and hybridization controls were comparable among samples. The background subtraction, expression summary, and normalization of gene signals were carried out using the robust multi-array analysis (RMA) method. For further normalization and analysis the expression signals were transformed to log-scale. The log-expression values were normalized such that the average of expression of those genes expressed in all samples was identical. Expression Fold Change (FC) for indicated genes is expressed as differences between Log2 normalized expression (treated cells) and Log2 normalized expression (untreated cells). Genes were filtered and analyzed using unsupervised hierarchical cluster analysis. Further processing included differential and functional analysis to identify the most significant biological processes and signaling pathways altered by LSESr treatment. For the differential analysis, the Benjamini and Yekutieli (BY) multiple comparison method was used to control the false discovery rate (FDR) in the analyses [47]. A *p*-value <0.05 after correction was considered significant. Gene Ontology (GO) was used to examine the common processes or underlying biological themes based on GSA (Gene Set analysis) and DAVID [48]. Comparison analysis of GO categories: Biological Processes, Molecular Functions and Cellular Components [49] for each time point as compared to untreated control was expected to identify the differentially expressed genes within a biological context.

### 3.6. Quantitative Real-Time PCR (RT-PCR)

Five hundred nanograms of total mRNA were processed to generate cDNA from random primers by reverse transcription using Superscript II (Life Technologies, Grand Island, NE, USA). RT-PCR was performed using SYBR green dye on an Applied Biosystems7900 Real-time PCR system (Applied Biosystems, Foster City, CA, USA). The following amplification conditions were used: 10 min at 94 °C, followed by 40 cycles of 30 s at 94 °C, and 30 s at 60 °C. The amount of each target gene related to the housekeeping gene Tata-binding protein (*TBP*) for each sample was determined using the comparative threshold cycle Ct method. The relative expression level of the target gene was calculated based on the difference in Ct values 2^−ΔΔCt^, between samples obtained from different treatment conditions and the reference sample representing untreated cells. Primer sequences are depicted in Table S2. Data were expressed as mean values (SD) derived from duplicate within each of three separate experiments. For Comparisons between groups, Non-parametric tests were applied in order to assess for potential differences between control and treatment conditions. *p* values <0.05 were considered significant. Statistical tests were performed by using GraphPad Prism 4.0 software (GraphPad Software: La Jolla, CA, USA).

## 4. Conclusions

By exploiting two *in vitro* cellular models of BPH, this study provided valuable insights into the potential anti-inflammatory role of LSESr in BPH management. At the same time, it continues to support the notion that inflammation is an important process in the pathogenesis of BPH. Further investigations are now necessary to determine the precise molecular basis of LSESr effects on inflammation in BPH. In addition, clinical studies using randomized, placebo-controlled, long-term trials are needed in order to test the anti-inflammatory effect of LSESr in human situations.

## Supplementary Information



## Figures and Tables

**Table 1:** List of selected genes significantly up-regulated or down-regulated at six hours of treatment with LSESr.

BPH1 cells				
Gene	Description	FC (MA *)	FC (qPCR **)	Representative GO TERMS
***CXCL6***	chemokine (C-X-C motif) ligand 6	−1.67	−0.94	inflammatory response, immune response, chemotaxis, defense response
***IL1A***	interleukin 1, alpha	−1.02	−1.49	inflammatory response, immune response, defense response, cell proliferation
***IL1B***	interleukin 1, beta	−1.13	−1.56	inflammatory response, immune response, defense response, cell proliferation, apoptosis
***IL1R1***	interleukin 1 receptor	−0.78	−1.29	immune response, defense response, cytokine-mediated signaling pathway
***PTGS2***	prostaglandin-endoperoxide synthase 2	−1.21	−1.67	fatty acid metabolic process, infllammatory response, cell poliferation, apoptosis
***ALOX5***	arachidonate 5-lipoxygenase	−0.58	−1.08	fatty acid metabolic process, defense response, inflammatory response
***PHLDA1***	pleckstrin homology-like domain, family A, member 1	−1.10	−1.04	apoptosis, cell death, glycoprotein metabolic process
***GAS1***	growth arrest-specific 1	1.45	1.79	cell proliferation, cell cycle arrest, apoptosis, cell morphogenesis
***DEDD***	death effector domain containing	0.77	0.36	apoptosis, cell death, transcription
***CDKN1B***	cyclin-dependent kinase inhibitor 1B (p27, Kip1)	0.50	NS	cyclin-dependent protein kinase inhibitor, apoptosis, proliferation, cell cycle arrest
**PrSF cells**				
***IL6***	interleukin 6 (interferon, beta 2)	−1.69	−1.83	inflammatory response, immune response, proliferation, chemotaxis, defense response
***IL8***	interleukin 8	−1.81	−1.91	inflammatory response, immune response, proliferation, chemotaxis, defense response
***NFkBIZ***	nuclear factor of kappa light polypeptide gene enhancer in B-cells inhibitor, zeta	−1.11	−1.32	inflammatory response, regulation of transcription, defense response
***TFRC***	transferrin receptor (p90, CD71)	−1.23	−1.77	endocytosis, vesicle-mediated transport, inflammatory response, defense response
***ERBB3***	v-erb-b2 erythroblastic leukemia viral oncogene homolog 3 (avian)	−0.97	−1.16	cell surface receptor linked signal transduction, apoptosis, proliferation
***JUN***	jun oncogene	−0.76	−1.81	apoptosis, cell death, proliferation, transcription
***CDKN1B***	cyclin-dependent kinase inhibitor 1B (p27, Kip1)	0.84	0.64	cyclin-dependent protein kinase inhibitor, apoptosis, proliferation, cell cycle arrest
***GAS1***	growth arrest-specific 1	0.73	NS	cell proliferation, cell cycle arrest, apoptosis, cell morphogenesis

* Log2 Fold changes from Microarray gene profiles were calculated as the difference between the log2 transformed normalized mean probe intensities from untreated *vs*. treated cells of three independent experiments;

** Log2 Fold changes from real-time qPCR as assessed using values from−(delta delta CT) derived from three independent experiments. Untreated conditions served as calibrator and TBP as reference housekeeping gene.

NS, not significant.
