# Supplementary material for: Lipidosterolic Extract of Serenoa Repens Modulates the Expression of Inflammation Related-Genes in Benign Prostatic Hyperplasia Epithelial and Stromal Cells
Source: Int J Mol Sci. 2013 Jul 10;14(7):14301–20. doi: 10.3390/ijms140714301 (PMC3742245; doi:10.3390/ijms140714301)
Supplement: Supplementary file 1 [file ijms-14-14301-s001.pdf]

Supplementary Information

Figure S1. Heat maps of significantly differentially expressed genes involved in proliferation (left) and apoptosis (right) GO terms in BPH1 cells (**a**) and PrSF cells (**b**) treated by LSESr at different time-points as compared to control.

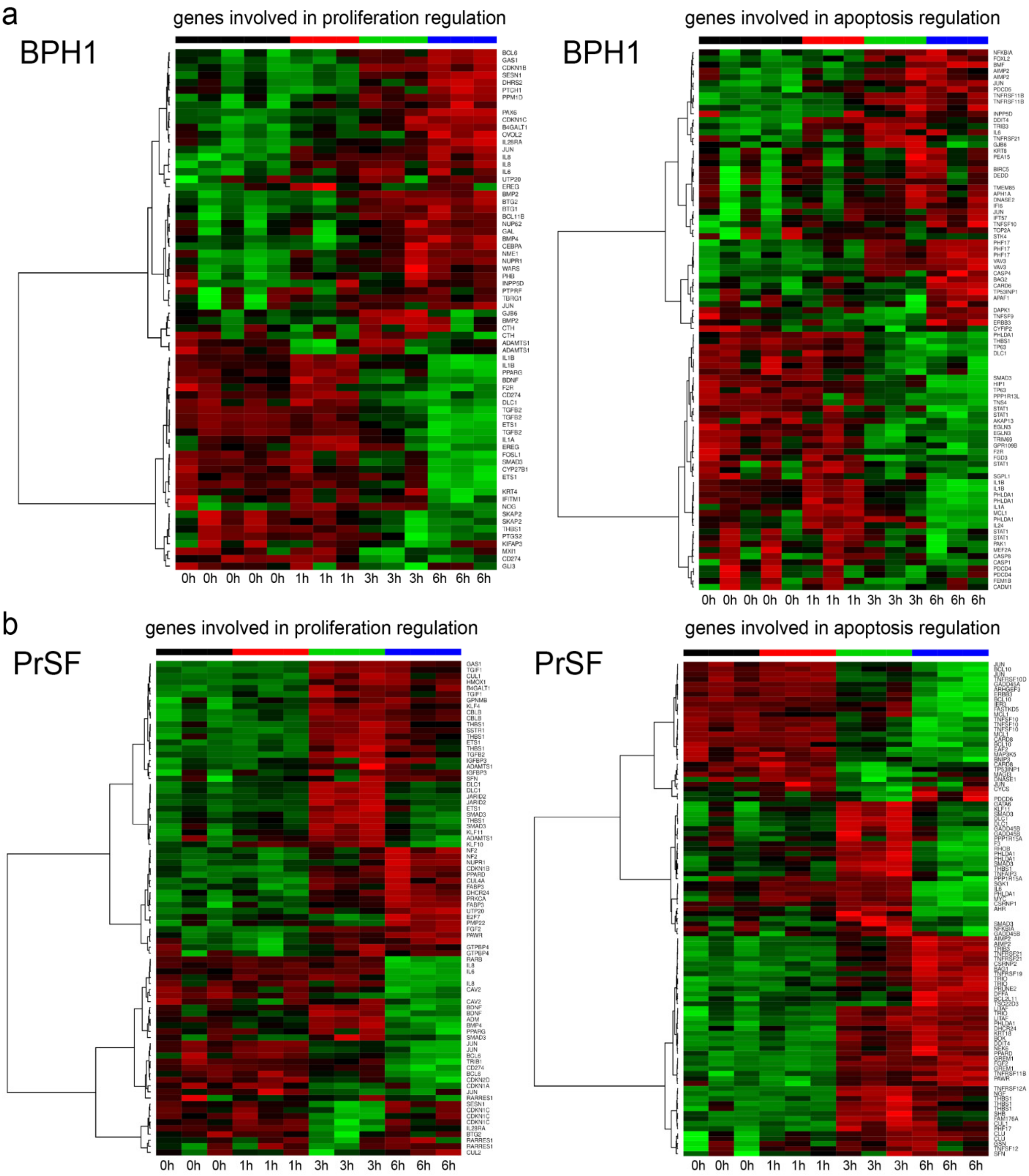

© 2013 by the authors; licensee MDPI, Basel, Switzerland. This article is an open access article distributed under the terms and conditions of the Creative Commons Attribution license (http://creativecommons.org/licenses/by/3.0/).
